# Applications and Research Advances in the Delivery of CRISPR/Cas9 Systems for the Treatment of Inherited Diseases

**DOI:** 10.3390/ijms241713202

**Published:** 2023-08-25

**Authors:** Xinyue Lu, Miaomiao Zhang, Ge Li, Shixin Zhang, Jingbo Zhang, Xiaoge Fu, Fengying Sun

**Affiliations:** Key Laboratory for Molecular Enzymology and Engineering of Ministry of Education, School of Life Sciences, Jilin University, Changchun 130012, China; xinyuel21@mails.jlu.edu.cn (X.L.); zhangmm21@mails.jlu.edu.cn (M.Z.); lige21@mails.jlu.edu.cn (G.L.); shixin20@mails.jlu.edu.cn (S.Z.); jbzhang22@mails.jlu.edu.cn (J.Z.); fuxg22@mails.jlu.edu.cn (X.F.)

**Keywords:** non-viral vectors, gene editing, nano delivery system, CRISPR/Cas9, viral vectors

## Abstract

The rapid advancements in gene therapy have opened up new possibilities for treating genetic disorders, including Duchenne muscular dystrophy, thalassemia, cystic fibrosis, hemophilia, and familial hypercholesterolemia. The utilization of the clustered, regularly interspaced short palindromic repeats (CRISPR)-CRISPR-associated protein (Cas) system has revolutionized the field of gene therapy by enabling precise targeting of genes. In recent years, CRISPR/Cas9 has demonstrated remarkable efficacy in treating cancer and genetic diseases. However, the susceptibility of nucleic acid drugs to degradation by nucleic acid endonucleases necessitates the development of functional vectors capable of protecting the nucleic acids from enzymatic degradation while ensuring safety and effectiveness. This review explores the biomedical potential of non-viral vector-based CRISPR/Cas9 systems for treating genetic diseases. Furthermore, it provides a comprehensive overview of recent advances in viral and non-viral vector-based gene therapy for genetic disorders, including preclinical and clinical study insights. Additionally, the review analyzes the current limitations of these delivery systems and proposes avenues for developing novel nano-delivery platforms.

## 1. Introduction

**Figure 1:**
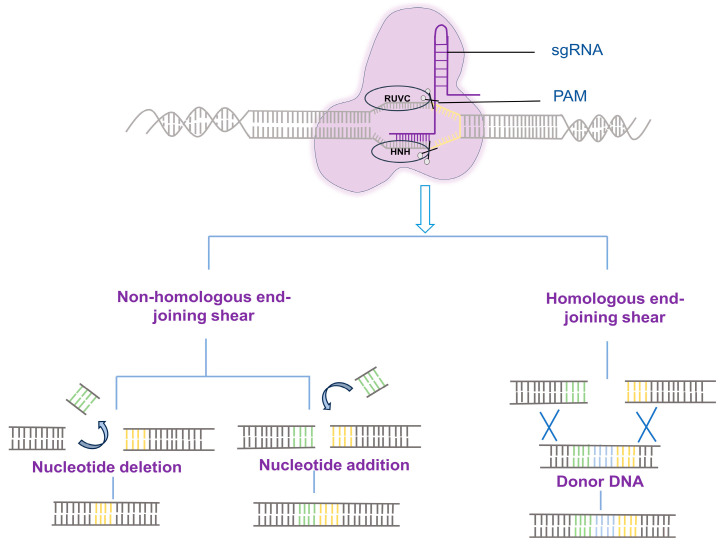
Repair Mechanisms for DNA fragments following CRISPR/Cas9 gene editing.

The development of gene editing has been accompanied by an increasing number of gene editing tools, including, but not limited to, zinc finger nucleases (ZFNs), transcription activator-like effector nucleases (TALENs), clustered, regularly interspaced short palindromic repeats (CRISPR)-CRISPR-associated protein (Cas) system. CRISPR/Cas system is a comprehensive, adaptive immune system in bacteria and archaea [1,2]. Researchers have utilized this system for precise genome editing by inducing double-strand breaks (DSBs) at specific sites, followed by repair via non-homologous end joining (NHEJ) or homology-directed repair (HDR) mechanisms, which allowed the insertion of desired DNA fragments [3]. The most commonly used CRISPR/Cas system is type II, which includes the DNA endonuclease Cas9, CRISPR RNA (crRNAs), and trans-activating crRNAs (tracrRNA). CrRNA and tracrRNA are fused to create a single guide RNA (sgRNA) upon application [4,5]. Although the CRISPR-Cas9 system has been extensively applied for genome editing in diverse organisms, including humans, animals, plants, and microorganisms, it faces challenges such as limited efficiency of homologous recombination and the risk of DSB-induced genetic abnormalities, such as chromosome translocations and rearrangements, which hinder its therapeutic utility for treating disease (Figure 1) [6].

In 2016, David Liu et al. introduced the base editor (BE) system, which advances gene editing without needing DNA double-strand breaks. This innovation garnered considerable interest and extensive adoption [7]. nCas9 was combined with the reverse transcriptase MMLV-RT in 2019, leading to prime editing (PE) development caused by exceptional editing (PE). PE promotes the efficient conversion of all 12 single nucleotides independent of DNA templates using an engineered guide RNA. In addition, it reduces the accurate insertion and deletion of multiple nucleotides [8]. However, both BE and PE are restricted to modifying a single base. Therefore, developing tools capable of site-specific integration of foreign DNA fragments without needing a double-strand break (DSB) remains an important field of research in genome editing.

David Liu and his colleagues created a new genome editing system called TwinPE by adding a site-specific serine integrase enzyme. This is an improvement over the previous PE system. TwinPE is designed to improve polyethylene (PE) performance and capabilities. This system employs a prime editing guide RNA (pegRNA) to introduce the integrase recognition site to the integrase recognition site. This system operates an excellent editing system for pegRNA. It uses a serine integrase to teach DNA sequences at the desired location and precisely introduce DNA sequences at the selected location. Notably, TwinPE enables the insertion of DNA fragments up to 40 kb in length with an approximate efficiency of 9.6%. It is essential to acknowledge that the TwinPE system necessitates artificially introducing integral sub-quality [9].

Meanwhile, the Yin Lab developed the GRAND editing system, which uses a pair of pegRNAs to facilitate efficient gene fragment insertion. Notably, the reverse transcriptase template within pegRNAs is non-homologous to the target site but complementary, allowing for precise targeting of 20–250 bp gene fragments using DNA reverse transcription. However, the efficiency of inserting a 400-bp element using this system must be optimized and enhanced further [10,11].

CRISPR/Cas9, a novel gene-editing technology, has shown enormous potential for gene therapy. In clinical applications, the secure and efficient delivery of CRISPR/Cas9 remains a significant obstacle [12]. Due to their biocompatibility, safety, and adaptable design, nanoparticles, including lipid-based, polymeric, gold, and virus-like nanoparticles, have emerged as potential delivery systems for CRISPR/Cas9-based gene therapy. These nanoparticles present novel delivery solutions for CRISPR/Cas9-based gene therapies. This article briefly overviews the application of various gene therapy delivery vehicles [13].

In summary, as the exploration of CRISPR/Cas9 research continues, a key challenge remains the successful delivery of the system into target cells. Viral vectors: lentiviral (LV), adeno-associated virus (AAV), adenovirus (AdV); non-viral vectors: virus-like nanoparticles (VLP), liposome (Lipo) and exosomes, along with certain physical methods, have been developed for delivery of CRISPR/Cas9 systems (Table 1). While viral vectors have limitations such as limited cargo capacity, potential genomic integration, and immunogenicity concerns, non-viral vectors have emerged as a solution, effectively overcoming these obstacles and enabling successful delivery of the CRISPR/Cas9 system into cells, resulting in impressive gene editing outcomes.

Even after addressing challenges related to CRISPR delivery, the efficiency of in vivo gene editing still falls short of expectations. Researchers and scholars contend that a substantial challenge for the future of gene editing lies in enhancing the accuracy and precision of the process. Confronting the challenge, scientists have continuously refined and advanced gene editing tools, resulting in a variety of editing tools characterized by heightened efficiency, precision, and reduced off-target effects(Table 2). The utilization of these sophisticated tools can increase the concentration of CRISPR delivered into the body, thus improving editing efficiency. It is anticipated that the following tools will find application in clinical contexts in the future, ushering in a promising era for gene therapy.

## 2. CRISPR-Associated Transposase

Transposons, also known as transposable elements, are widespread in prokaryotic and eukaryotic genomes. These genetic elements can relocate within the chromosome, leading to an increase in their copy number through a process known as transposition [40]. Various diseases caused by gene mutations or deletions can be treated using transposon-based targeted insertion systems. These systems enable precise integration and deletion of genetic material within the genome, independent of the repair mechanism of the host cell. This method dramatically expands the scope of genome editing in various cell types [41]. In addition, transposon-based strategies offer inherent advantages in terms of safety and efficacy compared to current methods for editing based on homologous recombination [42].

Several research groups have investigated improving transposon systems’ specificity by combining DNA-binding proteins with transposases. Cas9 has gotten much more attention than DNA-binding zinc finger or transcriptional activator-like effector proteins because it binds to its targets very strongly and stays there for a long time. Bhatt et al. used dCas9, a form of Cas9 that doesn’t have catalytic activity, to target the mariner transposon Hsmar1 for DNA integration [43]. While this model system was highly influential in vitro, it displayed an unusually high rate of random integration in *E. coli*. Similarly, when the Himar1 transposase was fused with the dCas9 nuclease, the engineered Himar1-dCas9 system achieved up to 80% site-specific plasmid insertion in *E. coli*.

Strecker et al. have devised a cyanobacterial CRISPR-associated transposase (CAST) system that permits targeted DNA insertion. The CAST system uses Cas12k and successfully targets the supplementary region of the 24 bp-spaced adjacent motifs (PAM), but it lacks cutting activity. TnsB and TnsC recognize and cleave specific sequences within the transposase-related donor DNA components, while TniQ is responsible for steering DNA insertion. CAST-mediated DNA insertion exhibits a directional preference; however, DNA fragment size and target site influence transposition efficiency. The off-target insertion rate, approximately 50%, must be considered. In contrast, Klompe et al. have developed the INTEGRATE system for targeted transposon insertion using gRNA [44]. The INTEGRATE system is better than the CAST system for inserting the same DNA fragment into multiple genome targets. Most of this system’s deposition occurs 80–82 bp downstream of the 5′-CC PAM, and the off-target insertion rate is lower (5%). Notably, DNA insertion in the INTEGRATE system is non-directional, and the transposition efficiency for 3–10 kb DNA fragments is lower than that of the CAST system.

The CRISPR-Cas systems, related to transposons, allow for efficient and substantial insertion of donor DNA at specific loci. This keeps the host cell’s repair mechanisms from being limited. However, it is necessary to address the particular values of these systems. At first, the transposon end sequences put into the genome along with the inserted DNA could make it hard to edit genes or insert DNA without leaving a scar. Even though these systems have shown that they can insert DNA into a few prokaryotic genomes, they still need to be improved to insert DNA into mammalian cells or model organisms. To be genuinely pertinent to disease treatment, they require further development.

Studies have revealed that PiggyBac (PB) and Sleeping Beauty (SB) transposons exhibit robust transposition activity in mammalian cells [45]. PB transposon excisions do not leave a CAG footprint and prefer local hopping, which limits their application. However, they have higher transposition activity and do not leave footprints after excision, which provides new gene-editing applications [46,47]. During transposition, the inverted terminal repeat sequences (ITRs) at both ends of the transposon vector are recognized by the PB transposase (PBase). Moving the target gene from its original location to the TTAA chromosomal site makes it more accessible. PB is a highly desirable instrument due to its high efficiency, safety, stability, security, and peace. 

Marc Guell et al. devised a gene delivery tool named FiCAT by integrating the targeting capability of CRISPR-Cas9 with the cutting and transferring functions of PB [48]. By disrupting the transposase recognition site during insertion, FiCAT makes translation irreversible. This innovative method permits the precise and targeted insertion of large DNA fragments between 2.5 and 9.5 kb. In humanHEK293 and mouse C2C12 cells, experimental results demonstrated that the FiCAT system attained a targeted insertion efficiency of approximately 25%. However, targeted insertion efficiency was diminished to 4% in the mouse liver and germ cells.

## 3. Disease Modeling and Gene Therapy

### 3.1. Duchenne Muscular Dystrophy

Duchenne muscular dystrophy (DMD) is an X-linked recessive genetic disorder characterized by dystrophin (Dys) gene mutations in the dystrophin (Dys) gene that eliminate dystrophin protein expression and destabilize muscle membrane structure [49,50]. Approximately 1 in 3000 to 1 in 5000 male young children are born with this fatal genetic disorder [51]. China has the highest prevalence of DMD, with about 400 to 500 cases reported annually and 70,000 to 80,000 infected individuals [52]. The 14 kb *Dys* gene sits between XP21.1 and XP21.2 and encodes the 427 kDa dystrophin protein [53]. There are about 7000 known mutations in the *DMD* gene. 70% of deletions and duplications occur in the central hot region encompassing exons 44 to 51, and the remaining 30% occur in the 5′ end deletion hot region containing exons 2 to 20 [54]. Point mutations are casually dispersed without an identifiable pattern throughout the gene [55].

Currently, there is no definitive treatment for DMD. Although steroid hormone therapy can delay the loss of ambulation and enhance the quality of life in patients with DMD, it is associated with adverse effects such as obesity, growth retardation, and osteoporosis and does not offer a cure. Due to how quickly genetic technology changes, using the CRISPR/Cas system to insert the *Dys* gene in a specific place has gotten much attention as a possible way to treat DMD [56]. In a preclinical canine model of DMD, Niclas E. Bengtsson et al. recently conducted a comparative study of two gene therapy approaches: gene editing and gene replacement. The results demonstrated that the gene replacement strategy led to more pronounced protein expression and greater improvements in muscle pathology compared to the gene editing approach. Furthermore, the therapeutic efficacy at the time of intervention was found to be correlated with the muscle pathology status. The study also highlighted the significance of age in the treatment of DMD. It was discovered that treatment efficacy varied with age, and the muscle pathology status at the time of intervention played a critical role in determining the treatment outcomes [57].

In a recent study conducted by Tatiana V. Egorova and her colleagues, a new dual-cleavage strategy using the CRISPR/Cas9 system was employed to remove exons 6 and 7 of the *Dys* gene and correct the reading frame of the myotonic dystrophy protein in a DMD mouse model. Sanger sequencing was able to correct mRNA expression by 30% to 50%, providing a novel method for diagnosing DMD [58]. Several approaches, such as antisense oligonucleotides (ASOs), gene therapies, and stop codon read-through drugs, have been either approved or are currently under investigation for treating DMD disease [59]. Despite some observed therapeutic effects, the transient nature of these approaches limits their clinical application. In contrast, the utility and accuracy of CRISPR/Cas9 make it a promising intervention with potential long-term benefits.

### 3.2. Hemophilia

Hemophilia is an inherited bleeding disorder characterized by the deficiency of *clotting factor VIII* (hemophilia A) or *clotting factor IX* (hemophilia B). This deficiency impairs the normal clotting process, leading to prolonged bleeding following injury. The severity of hemophilia varies based on the level of clotting factor in the blood. Severe hemophilia is associated with frequent spontaneous bleeding, whereas mild hemophilia typically manifests as bleeding following trauma or surgery.

Matsui, H. et al. demonstrated the efficacy of a non-viral vector gene delivery system involving the piggyBac DNA transposon in treating hemophilia A. The PB vector efficiently transfected HEK293T and iPS cells through in vitro electroporation, resulting in stable and sustained expression of FVIII for up to 300 days in vivo when administered via tail vein injection in mouse models. The treated group had significantly less mean hemorrhage time (6 min and 13 s) than the control group (18 min and 24 s). The PB vector proved to be an efficient tool for mediating the efficient and durable expression of the full-length *FVIII* gene, thereby enhancing the hemostatic profile of hemophilia A rodents [60].

Verma et al. conducted a study where they treated hemophilia B mice lacking *Factor IX* (*FIX*) using the LUNAR platform, a safe and reliable liposomal nanoparticle (LNPs) mRNA delivery system. This approach resulted in the rapid correction of coagulation abnormalities and exhibited therapeutic effects that lasted for 4–6 days. Notably, when delivering the fixed variant *R338A* mRNA, LUNAR LNPs demonstrated an impressive 8~10-fold increase in therapeutic efficacy and coagulation activity compared to adenoviral vectors. These findings underscore the immense potential of LUNAR as a platform for protein replacement therapy in diseases like hemophilia, offering enhanced therapeutic benefits [61].

Some academics and researchers have proposed innovative strategies for hemophilia gene therapy based on previous scientific research that highlighted the limitations of viral vectors in delivering CRISPR/Cas systems [62]. One such approach, undertaken by Jeong Hyeon Lee et al., introduced the *human Factor 9* (*hF9*) gene into the antithrombin gene using a combination of non-viral vector LNP and adenoviral vectors. The study demonstrated the successful reduction of antithrombin levels and the production of hFIX protein, leading to the restoration of coagulation function. Importantly, the intervention of LNP substantially amplified the knock-on effect of the gene in vivo and contributed to the reduction of AAVs used in the therapy. These findings lay a solid foundation for the development of genetic disease treatments involving gene editing and protein deficiency [63].

Qiyu Tang et al. investigated the pathophysiology of Hemophilia B (HB) and elucidated that the underlying cause of HB is a deficiency of functional coagulation *FIX*. However, a research model still needs to be developed. To achieve this, they conducted in vitro CRISPR/Cas9 integration of active *FIX* variants into human induced pluripotent stem cells (hiPSCs). The successful integration of the *FIX* variants was carefully screened and confirmed. Subsequently, *FIX* activity in the cell supernatant was quantified, revealing a remarkable increase of 4.2-fold compared to normal cells and a substantial enhancement of 63.64% above the average level. These findings offer valuable insights into the therapeutic mechanism of *HB* gene therapy and provide a new idea for investigating gene therapy for HB treatment [64].

### 3.3. Cystic Fibrosis

Cystic fibrosis (CF) is an autosomal recessive monogenic disorder that impacts an estimated population of 70,000 in the United States and Europe [65]. The mutation of the Transmembrane Conductance Regulator (CFTR) gene is the underlying cause of cystic fibrosis, which causes dysfunctional chloride ion transport. Given the genetic basis of the disease, genome therapies can target and correct specific alterations, thereby offering the possibility of long-term treatments for cystic fibrosis. The emergence of CRISPR/Cas9 as a gene editing instrument, coupled with advances in nuclease technology, has created new opportunities for precise genome correction and holds tremendous promise for treating cystic fibrosis and other genetic disorders [66,67].

Anna Cereseto et al. utilized a CRISPR-based adenine base editing (ABE) strategy to correct mutations without inducing DNA double-strand breaks (DSBs). Their research revealed a cellular modification efficacy of up to 70% [68]. CF gene therapy has been used in several animal models [69]. In an individual study, Katarina et al. attempted to improve Cas9-RNP delivery efficiency. They created a strategy based on the non-covalent attachment of ABE to the amphiphilic S10 shuttle peptide. In addition, they developed S315 as a penetrating peptide to facilitate the formation of ABE8e-Cas9 RNP and S315 vectors for efficient delivery in rhesus airway epithelia. This method achieved up to 5.3% gene editing efficacy [70].

The establishment of a rodent model for CF is crucial for investigating the underlying mechanisms of the disease and exploring potential therapeutic approaches. In this regard, Alexandra McCarron and her colleagues successfully developed a viable rodent model of cystic fibrosis, which offers promising opportunities for longitudinal assessments of CF pathophysiology and therapeutic interventions [71].

The straightforward CF model has proven to be valuable in gene editing experiments. In this context, researchers utilized the CF model to edit the EGFP and CFTR genes in ferret airway basal cells through the use of the rAAV vector. Notably, green fluorescence sorting of CFTR gene-edited cells revealed a successful editing rate of 70.4% among the cells, while the CFTR gene correction rate was 3%, and the EGFP gene correction rate was 18.2%. These findings strongly indicate that the co-editing of two genes can enhance the efficiency of gene editing through homology-directed repair [72].

### 3.4. Thalassemia

β-thalassemia is a genetic disorder that mainly impacts children, resulting in delays in growth and complications, including chronic hepatitis, cirrhosis, and osteoporosis. Historically, the prognosis for β-thalassemia patients has been dismal, with high mortality rates caused primarily by cardiac disease [73]. However, with the advent of gene therapy, there is renewed optimism for a potential genetic solution for transfusion-dependent β-thalassemia (TDT). TDT and sickle cell disease (SCD) are the most prevalent monogenic disorders worldwide, with approximately 60,000 new cases of TDT and 300,000 new cases of SCD each year [74]. These diseases are characterized by mutations in the *hemoglobin beta subunit gene* (*HBB*), which encodes adult hemoglobin, the primary oxygen-carrying protein in red blood cells [75]. Hemoglobin comprises α-globin and β-globin chains [76], with alterations in hemoglobin synthesis leading to thalassemia disorders [77].

Currently, the only approved and effective treatment for β-thalassemia is allogeneic transplantation of hematopoietic stem cell (HSC), but there are issues, such as potentially inadequate donor histocompatibility [78]. Clinical trials have attempted to introduce the functional hemoglobin *HBB* gene into HSC, but concerns regarding biosafety have arisen due to ex vivo viral transfection, hindering clinical translation [79]. To address this, Qian Ban et al. proposed an alternative approach by utilizing the CRISPR/Cas9 system delivered through a supramolecular nanoparticle (SMNP) vector to activate the *HBB* gene. The vector was directly introduced into the bone marrow of rodents, leading to the successful integration of the *HBB* gene into the genomic DNA locus of hematopoietic stem and progenitor cells (HSPC). This innovative strategy offers a promising avenue for treating β-thalassemia [80].

Single-gene mutations in HSC subpopulations are a significant contributor to thalassemia. Shuqian Xu et al. proposed a therapeutic strategy aimed at effectively repairing HSC to combat the disease. By utilizing Cas9 RNP, they successfully modified the genes of primary hematopoietic stem cells obtained from thalassemia patients. This gene editing approach rectified the issue of abnormal splicing and restored the expression of globin, addressing the fundamental defect in thalassemia. The success of this corrective strategy offers a promising research foundation for the development of innovative treatments for thalassemia [81].

*BCL11A* is a transcription factor essential in modulating the expression of globin and fetal hemoglobin in erythroid cells. The *BCL11A* erythroid-specific enhancer has been targeted with CRISPR-Cas9 technology, effectively modifying approximately 80% of the alleles at this specific genomic locus. Notably, no off-target editing events were observed, showing the high specificity of the CRISPR-Cas9 strategy [82]. In a study by Bin Fu et al., autologous hematopoietic stem and progenitor cells with edited *BCL11A* enhancers were introduced into the body. This strategy yielded extraordinary results, with patients attaining independence from transfusions for more than 18 months. Moreover, the edited cells displayed high levels of gene editing efficiency, with 85.46 and 89.48% of cells exhibiting the desired modifications, respectively. Notably, no adverse effects were observed, emphasizing the safety and efficacy of this gene editing strategy [83].

### 3.5. Familial Hypercholesterolemia

Beginning at birth, familial hypercholesterolemia (FH) is a genetic disorder characterized by elevated levels of low-density lipoprotein (LDL) cholesterol in the blood. This condition carries a substantial risk of developing cardiovascular disease (CVD) over time [84]. FH is a common inherited hyperlipidemia in children. It is one of the most severe lipid disorders linked to cardiovascular complications and a significant coronary artery disease risk factor [85]. Previous studies have estimated the frequency of heterozygous FH to be approximately 1:500, although recent data suggest that the prevalence may range from 1:200 to 1:300 [86,87]. In 2003, the role of the *PCSK9* protein in lipid metabolism was discovered [88]. *PCSK9* binds to the LDL receptor on the surface of hepatocytes. This stops the receptor from recycling and keeps it from coming back to the cell surface [89]. LDL particles are typically removed from the circulation by adhering to hepatocyte LDL receptors [90].

FH is a group of genetic disorders of lipoprotein metabolism characterized by severe hypercholesterolemia and LDL receptor (LDLR) deficiency [91]. Existing drugs for the treatment of FH, such as statins and *PCSK9* inhibitors, have shown limited efficacy, particularly in cases of HoFH. On the other hand, viral-mediated *LDLR* gene transfer therapy has demonstrated insufficient durability. As a result, there is a critical need to explore alternative treatments that can effectively restore LDLR function. Hirofumi Okada et al. used induced pluripotent stem cells (iPSC)-differentiated hepatocyte-like cells (HLCs) as a model of FH. They utilized CRISPR/Cas9 gene editing to achieve long-term and efficient correction of the LDLR [92,93]. Additionally, the method’s immunogenicity was thoroughly investigated, including its impact on the patient’s peripheral blood mononuclear cells (PBMCs). This comprehensive research provides a solid foundation for the potential clinical application of gene therapy as a promising treatment approach for FH [94].

Lentiviruses outperformed adenovirus (AAV), which has been recognized as an effective vector for treating the FH mouse model. Nevertheless, preclinical research in large animal models is necessary. In the WHHL rabbit model, Elisa Hytönen et al. showed that lentiviral vector-mediated *LDLR* gene transfer significantly reduced cholesterol levels [95]. Surprisingly, AAV vectors in the WHHL rabbit model provide a valuable foundation for the clinical development of efficient and secure delivery vectors [96].

Julius L. Katzmann and his colleagues used a CRISPR method based on an adenoviral vector to target the *PCSK9* gene. They aimed to disrupt certain regions and lower the amount of *PSCK9* protein. Their study showed a 9% editing efficiency, indicating the potential for FH gene therapy [16]. FJ Real et al. thought that Evinacumab, an anti-ANGPTL3 monoclonal antibody, could treat people with homozygous FH. Numerous studies have demonstrated that Evinacumab can significantly lower LDL cholesterol in people with the illness by 47.1% [97]. Vanhoyo et al. investigated familial hyperlipoproteinemia (FHBL), which results from an early stop codon in the *APOB* gene. They were able to make *APOB* knockout (KO) knock-in Huh9 cells and *APOB* knockout (KO) knock-in Huh9 cells. They then used ELISA and PCR to measure the amount of APOB in the cells. While *APOB* expression was 70% lower in heterozygous APOB-KO cells, it was almost nonexistent in homozygous APOB-KO cells. This work highlights CRISPR/Cas9’s capability to treat FHBL [98].

### 3.6. Diabetic Retinopathy

The number of people affected by diabetic retinopathy (DR) and related visual impairment is expected to rise due to the projected sharp rise in diabetes worldwide [99]. DR is a microvascular condition that significantly worsens overall vision [100]. Although significant research has been done on its etiology, treatment options for DR are still limited despite substantial research on its etiology, with up to 50% of patients responding insufficiently to current medications [101].

Abnormal angiogenesis is an essential aspect of PDR disease, and the phosphoinositide 3-kinase (PI3K) signaling pathway plays a vital role in this process. Research has shown that the vascular membrane of a PDR mouse model exhibits high levels of the catalytic subunit of PI3Ks, *p110* [102,103]. Wu et al. targeted this pathway and developed a dual AAV system for CRISPR/Cas9 delivery. One set of AAVs was designed to knock down the p110 gene, while the other set targeted the intercellular adhesion molecule 2 (pICAM2) promoter. The final results demonstrated a significant decrease in pathological retinal angiogenesis and an effective reduction in *p110* expression. This novel strategy effectively modifies PI3Ks, offering a promising approach to address aberrant angiogenesis in the retina [104].

In a mouse model of DR damage, Axl, a receptor tyrosine kinase, has been identified as a key factor in preventing retinal angiogenesis. Wu et al. conducted experiments to investigate the role of Axl in human retinal microvascular endothelial cells (HRECs) exposed to PDR vitreous. By using gene editing techniques to limit Axl expression, they successfully inhibited the activation of Akt in HRECs. Additionally, Axl was found to indirectly initiate the intracellular process that leads to the production of vascular endothelial growth factor (VEGF-A), further indicating its significance in the development of PDR [105].

Ao et al. used the CRISPR/Cas9 strategy to knock down the *thioredoxin-interacting protein* (*TXNIP*) gene to study the role of autophagy and apoptosis in the therapy of DR. Their research shed light on the etiology of DR by showing the positive regulatory function of TXNIP in autophagy in rat Müller cells under hyperglycemic conditions [106]. Li showed that upregulating VE-calmodulin and lowering *NADPH oxidase 4* (*NOX4*) levels resulted from downregulating *CCN1* using siRNA and CRISPR/Cas9 technology. By activating *NOX4*, it has been found that elevated *CCN1* expression promotes oxidative stress and affects the integrity of tight junctions in endothelial cells. A potential therapeutic approach for treating DR entails concentrating on the *CCN1/NOX4* gene pathway to reduce endothelial cell damage [107].

## 4. Delivery of Biomacromolecules

### 4.1. Duchenne Muscular Dystrophy

#### 4.1.1. Adeno-Associated Virus (AAV)

**Figure 2:**
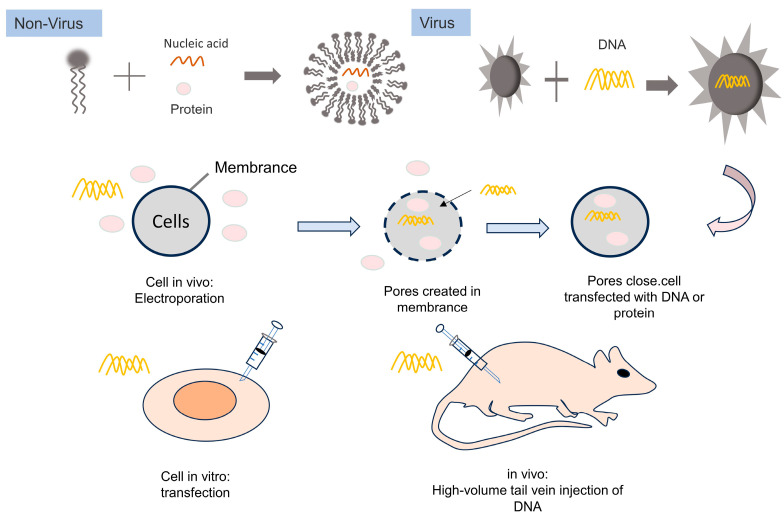
Gene editing strategies utilizing viral and non-viral vectors in vitro and in vivo.

Adeno-associated virus (AAV) has shown specific targeting capability for muscle cells, making it a potential carrier for the *Dys* gene. However, the limited packaging capacity of AAV vectors (up to 4.7 kb) restricts the delivery of full-length *Dys* genes, and only truncated *micro-Dys* that can produce partial proteins for gene replacement therapy can be accommodated [108]. While *micro-Dys* has demonstrated improved muscle function in mouse models of Duchenne muscular dystrophy (*MDX mice*) and Golden Retriever Muscular Dystrophy (GRMD), it is vital to consider the differences in muscle mass and atrophy severity between animal models and human patients. Consequently, micro-Dys’ efficacy in improving muscle function in humans remains uncertain. Challenges such as potential cellular toxicity, immunogenicity, and long-term expression of viral vectors hinder their clinical application [109]. AAV vectors integrate the carried genes into the host DNA, often close to genes involved in cell growth regulation. This raises concerns about potential genomic alterations that could contribute to liver cancer and pose a risk for oncogenesis (Figure 2).

Since DMD gene mutations are varied, several CRISPR/Cas9 gene editing strategies are required to treat all DMD patients completely. Therefore, it is crucial to investigate more accurate and effective endonuclease techniques to improve the safety and effectiveness of gene editing and hasten the development of full-scale treatments for DMD patients. AAV capsids with a 7-mer RGD motif have been identified by Tabebordbar et al. as belonging to the Myo AAV group. Following intravenous treatment in mouse and non-human primate studies, these capsids showed muscle-specific gene transport through integrin heterodimers following intravenous treatment in mouse and non-human primate studies. Furthermore, Weinman et al. reported finding mouse muscle-tropic capsid variants with RGD motifs [110].

#### 4.1.2. Lentiviral Vector (LV Vector)

LV vectors contain viruses with two single-stranded genomic RNAs of around 9 kb each. They have an approximate diameter range of 80 to 120 nm. Reverse transcription of these RNAs releases double-stranded DNA, which integrates into the host chromosomes [111]. LV vectors can handle gene segments of up to 8 kb better than AAV vectors because a cellular lipid bilayer surrounds them rather than a viral capsid. Due to this property, they are less immunogenic than AAV vectors [112]. The integration of LV vectors into the host genome and their continued gene expression slow the delivery of CRISPR-Cas9. This is because viral DNA’s random integration could turn oncogenes on and increase the risk of Cas9 mutagenesis in the wrong place [113].

Researchers have looked into ways to improve their safety profile to address the genotoxicity that LV vectors cause. A specific strategy delivers gene editing using integrase-deficient lentiviral vectors (IDLV) [114]. Uchida et al. successfully corrected mutations linked to sickle cell disease in the globin gene by delivering CRISPR-Cas9 components into human cord blood-derived erythroid progenitor cells (HUDEP-2) using IDLV vectors. However, it was recognized that using IDLV vectors still carries the danger of viral DNA integration [115].

### 4.2. Non-Viral Delivery of Genome-Editing Systems

#### 4.2.1. Exosomes

**Figure 3:**
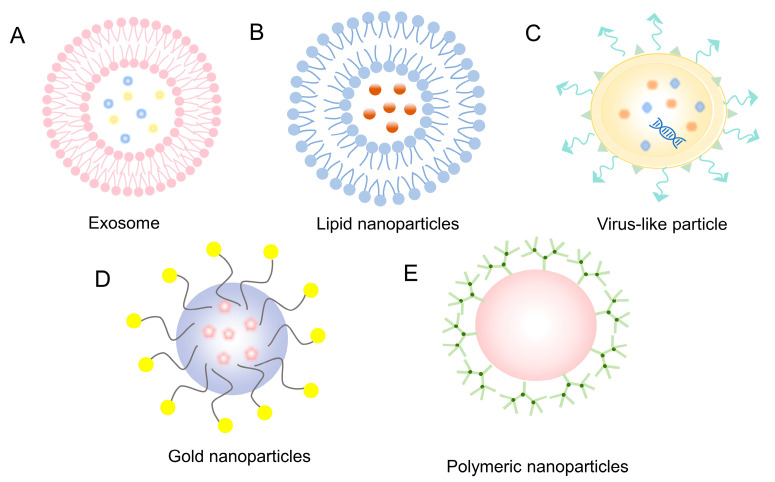
Non-viral delivery vectors for gene editing. (**A**) Exosomes; (**B**) Lipid nanoparticles; (**C**) Virus-like particle; (**D**) Gold nanoparticles; (**E**) Polymeric nanoparticles.

Exosomes are tiny, lipid membrane-bound nanovesicles with a diameter of between 30 and 150 nm that cells secrete [116]. They can be extracted from various extracellular fluids from cell cultures, such as blood, saliva, amniotic fluid, urine, and supernatants from cell cultures. Exosomes play an essential role in intercellular communication and have attracted considerable interest in clinical diagnostics and therapeutics [117]. Exosomes have emerged as promising vehicles for targeted drug delivery in recent years. However, their efficiency in encapsulating sizable nucleic acid fragments is relatively low [118]. Hybrid nanoparticles of exosomes and liposomes are better at enclosing larger plasmids, such as CRISPR/Cas systems linked to transposons [119]. Consequently, hybrid exosomes and liposome nanoparticles have tremendous potential for gene editing applications in vivo (Figure 3A).

#### 4.2.2. Lipid Nanoparticles

Numerous non-viral delivery mechanisms for efficient in vitro delivery of CRISPR/Cas9 have been developed recently. Among these methods, lipid nanoparticles (LNPs) have attracted significant interest as efficient carriers for transporting massive nucleic acid and protein payloads [120]. LNPs can enclose different ways to edit the genome, such as Cas9 mRNA, Cas9 protein, CRISPR/Cas9 RNPs, and base editors, which makes it possible to edit genes in cells in a targeted way [121]. These LNPs offer many advantages, including biodegradability, biocompatibility, and protection of genome-editing systems [122]. In addition, LNPs are easily modifiable to improve delivery efficiency and accomplish cell- or tissue-specific targeting (Figure 3B).

#### 4.2.3. Virus-like Particle

In the context of lentivirus-based delivery systems for gene-editing therapies, researchers have recognized the associated risks and have developed Virus-like particles (VLPs) as an alternative approach (Figure 3C). VLPs are self-assembled structures composed of structural proteins, resembling natural viruses but lacking a viral genome, thereby preventing replication within host cells [123]. Recently, VLPs have garnered significant attention, particularly in vaccine development [124]. Liu Qi et al., for instance, devised a virus-like vector for co-delivering the CRISPR-Cas9 system and small-molecule medications for the curative use of malignant tumors. Their VLP construct demonstrated structural stability in the circulation, and western blot analysis revealed a 45.1% gene editing efficiency, providing valuable insights for using VLPs to deliver CRISPR systems for the treatment of disease [125].

#### 4.2.4. Gold Nanoparticles

Since they do not react with chemicals and are safe for the immune system, gold nanoparticles (AuNPs) with diameters between 1 and 100 nm have gotten much attention as delivery vehicles for gene editing systems (Figure 3D) [126]. Their low toxicity and high efficacy make them a promising alternative for safely delivering genes [127]. The size and intracellular retention time of AuNPs influence their effectiveness as in vivo therapeutic delivery vehicles [128,129]. Shahbaz et al. created colloidal AuNPs to deliver the CRISPR/Cas9 plasmid DNA system, demonstrating low toxicity and high editing efficiency in hematopoietic stem cells [130].

Wang Peng et al. reported a strategy for using a multifunctional vector to deliver the Cas9-sgPIk-1 plasmid (CP) for tumor therapy. Electrostatic interactions have been used to assemble CP onto TAT peptide-modified AuNPs to produce a LACP complex. The TAT peptide was used for nuclear targeting to reduce tumor gene expression. This strategy effectively lowered the melanoma target gene PIK-1, resulting in a 65% decrease in protein expression, and demonstrated significant tumor suppression in animal models [131].

#### 4.2.5. Polymeric Nanoparticles

As drug delivery vehicles, polymeric nanoparticles have several advantages, such as their small size, controlled drug release, ability to break down in the body, and lessening immunogenicity (Figure 3E) [27]. CRISPR/Cas9 systems encased in polymeric nanoparticles can work better by changing their functions [132,133]. PEI is a hydrophilic, cationic polymer that electrostatically binds to negatively charged DNA, facilitating endosomal escape [134]. Ryu et al. used PEI to get CRISPR/Cas9 into Neuro2a cells. They had over 70% transfection efficiency and around 20% insertional deletion efficiency. However, the high cationic charge of PEI can lead to increased toxicity, limiting its application in vivo and in clinical settings [135]. Abbasi et al. also made poly (ethylene glycol) polymer micelles that enclose Cas9 mRNA and sgRNA for efficient delivery to cells and protect sgRNA from being broken down by enzymes. They demonstrated in vivo gene editing using these multimeric micelles in animal models [136].

## 5. Current Status of Genome-Editing Clinical Trials

The CRISPR system has emerged as a prominent gene-editing tool in clinical research, demonstrating its potential in various applications, including vital gene screening, cancer immunotherapy, and the treatment of genetic diseases [137]. CRISPR/Cas technology has been widely used for genome editing in bacteria, plants, animals, and humans, allowing for the rapid generation of disease models and therapeutic strategies for human genetic disorders [138,139]. Animal models, like mice, rats, piglets, and non-human primates, have been used to study gene editing with sgRNA, Cas9, or mRNA both in vivo and in vitro [140]. However, the susceptibility of free sgRNA to nucleases limits its therapeutic effectiveness [141]. Researchers have focused on making new sgRNA delivery vectors, including viral and non-viral vectors, to improve the efficacy of gene delivery for both in vivo and in vitro applications [142,143]. This has made gene editing a more promising therapy.

In recent years, gene therapy for Leber’s congenital amaurosis (LCA), a rare inherited retinal disease, has made significant progress. The FDA approved Luxturna’s marketing approval in December 2017 [144]. Notably, in 2020, an 8-year-old child with LCA treated with Luxturna demonstrated significant improvement in vision [145]. For treating LCA10, a clinical trial employing CRISPR gene editing technology is currently underway, and preliminary results were recently published. In addition, over 40 genetic medicines are currently in clinical trials for treating ocular genetic diseases in vivo [146].

In the clinical management of thalassemia, the two most prevalent gene editing strategies are the induction of fetal hemoglobin (HbF) expression and the precise repair of mutations in the HBB gene [147]. In August 2022, Wu Yuxuan’s East China Normal University team conducted a Phase I/II clinical trial with two children who achieved independence from blood transfusions for up to 16 months after transplantation. Notably, there were no significant adverse effects observed [148]. Ongoing research is enhancing the adaptability of the CRISPR system and investigating gene editing tools for treating other diseases, including liver diseases [149], are also underway. Although gene editing clinical trials for inherited diseases are still in their infancy, the unique pathogenesis of these conditions makes CRISPR/Cas9 a promising and soon-to-be-available strategy for treating inherited diseases.

## 6. Conclusions and Future Directions

The advent of CRISPR/Cas gene editing technology has significantly accelerated the development of gene editing therapies. Extensive preclinical studies have shown that CRISPR/Cas9 and its offshoots, such as single-base and bootstrap editors, can accurately remove or fix mutations in individual genes that cause disease. In addition, these technologies can be used to modify disease-associated genes, which may have therapeutic applications. Numerous clinical trials employing CRISPR/Cas-based genome editing to treat various genetic diseases and cancer have been approved, with in vivo applications making limited progress. However, ongoing research bears promise for the potential cure of certain genetic disorders within the next 5–10 years using gene editing therapies.

Developing engineered nucleases, such as ZFN/TALENs and CRISPR/Cas9, has paved the way for translating gene editing concepts into clinical practice. In the future, CRISPR systems are anticipated to find more extensive applications in treating diverse diseases, particularly cancer. In addition, gene editing technologies have facilitated advances in cellular anti-aging, genetic diagnostics, and the creation of therapeutic medications. Although off-target effects are still a concern in gene editing, advancements in gene delivery vectors have increased delivery efficiency and decreased toxicity, bringing gene editing closer to clinical application. Genome editing has the potential to reveal the underlying biological mechanisms underlying disease development and progression through sustained research and international scientific collaboration, thereby offering novel therapeutic approaches and advancing the field of life sciences.

## Figures and Tables

**Table 1:** Past Limitations of CRISPR and research updates.

Delivery Strategies	Delivery Approach	Limitations	Advantages	Applications	References
LV	CRISPR/Cas9 and sgRNA	LV vectors are at risk of off-target mutations and have a limited loading capacity of 10 kb bases.	It can deliver CRISPR land to cells in a single transfection and has a high cloning capacity. Low immunogenicity and inexpensive expansion	In vitro	[14,15]
AAV	CRISPR/Cas9	Includes a 4.7 kb fragment that readily integrates into the host’s genome.	AAV capsids are structurally flexible, serotype-diverse, and easily adaptable to suppress the immune response.	In vitro and in vivo	[16,17,18]
AdV	CRISPR/Cas9	Packaging restricted to 8 kb fragments, prone to adverse immune reactions, more challenging to prepare	Lower risk of off-target effects and insertion mutagenesis, together with better clinical outcomes	In vivo	[19,20]
VLP	RNA	Limited clinical translation, instability, and insufficient support for widespread use	Excellent biosecurity, low immune response, and flexibility	In vivo	[21,22]
Lipo	CRISPR/Cas9 DNA	High storage and transport requirements, limited DNA concentration at delivery	High load efficiency, editing security, efficiency, and specificity	In vivo	[23,24]
Exosome	CRISPR/Cas9 DNA	Complex preparation, extreme storage and transport conditions, and susceptibility to degradation	Natural targeting ability, reduced immune response, and excellent biosecurity	In vivo	[25,26]
Polymer-based	CRISPR/Cas9 sgRNA	Possible to aggregate, destabilize, and be eliminated from the organism.	Small size, controlled release, biodegradable, lower immunogenicity	In vivo	[27]
Inorganic nanoparticles	CRISPR/Cas9	Slow degradation in vivo, simple hepatic accumulation, and specific toxicity in vivo	Small size, sizeable small size, high effectiveness, delayed controlled release, targeted action, and the ability to escape an organelle called	In vivo	[28,29]

**Table 2:** Challenges and improvements in CRISPR and editing tools.

Gene Editing Tools	Off-Target Risk	Improvement	Gene Type	Clinical Application	Reference
ZFN	High	Optimization of DNA structural and catalytic domains using the modular structure of ZFNs	DNA	Hemophilia B and β-Thalassemia proceeded to clinical stages I and II, respectively.	[30,31,32]
TALEN	High	High-throughput solid-phase assembly, connection-independent cloning, and “Golden Gate” molecular cloning are just a few examples.	DNA	Clinical Phase I in HPV-related cervical intraepithelial neoplasia	[33,34]
CRISPR/Cas9	Moderate	Improved targeting to the interior of the nucleus and increased mRNA stability	DNA	β-Thalassemia clinical Phase II	[35]
CRISPR/Cas13	Low	Figuring out whether an RNA substrate binding site exists at the catalytic site of the Cas13 protein	single-stranded RNA	Proceed to preclinical studies	[36,37]
BE	Moderate	Enhancing their sequence preferences and coming up with methods to efficiently assess off-targeting	DNA	Numerous studies have laid the groundwork for conducting clinical	[38]
PE	low	It enhanced PE in various cells and organisms to evaluate off-target effects across the genome.	pegRNA	No clinical studies have been conducted at this time	[8]
TwinPE	Low	The effectiveness of gene editing is significantly boosted by adding two pegRNAs on top of PE.	Paired pegRNA	No clinical studies have been conducted at this time	[39]

## Data Availability

Not applicable.
